# Prevalence of the metabolic syndrome in Pudong New Area of Shanghai using three proposed definitions among Chinese adults

**DOI:** 10.1186/1471-2458-10-246

**Published:** 2010-05-12

**Authors:** Wang-hong Xu, Xiao-nan Ruan, Xiao-jin Fu, Qiu-li Zhu, Hong Zhang, Yun Bai, Hong-yan Wu, Yi Zhou, Hua Qiu, Qiao Sun, Qing-wu Jiang, Li-ming Yang, Jian-jun Gu, Gen-ming Zhao

**Affiliations:** 1 Department of Epidemiology, School of Public Health, Fudan University; Key Laboratory of Public Health Safety, Ministry of Education (Fudan University), 138 Yi Xue Yuan Road, Shanghai 200032, China; 2 Pudong New Area Centers for Disease Control and Prevention, 3039 Zhang Yang Road, Shanghai 200136, China; 3 Health Bureau of Shanghai Pudong New Area, 820 Cheng Shan Road, Shanghai 200125, China

## Abstract

**Background:**

The prevalence of metabolic syndrome (MS) has been increasing in China in recent years. The aim of this study is to estimate and compare the prevalence of MS among Chinese adults in Shanghai, one of the most economic developed areas in China, using definitions proposed by World Health Organization (WHO), National Cholesterol Education Program Adult Treatment Panel (modified ATP III) and International Diabetes Federation (IDF).

**Methods:**

This cross-sectional study included 5,584 adults at age 20-79 randomly selected from Pudong New Area of Shanghai, China, through a three-stage sampling. All participants were interviewed in-person between April and July of 2008 to collect information on demographic and lifestyle characteristics. At the interview, anthropometry and blood pressure were measured and bio-specimens were collected.

**Results:**

The prevalence estimates for the MS increased with age for each definition in men and women, but the estimates varied greatly between the definitions and by sex. The prevalence of the MS was higher in men (20.2%) than in women (18.7%) using WHO definition but this sex difference was reversed when using the modified ATP III (28.4% for men vs. 35.1% for women) and the IDF (15.9% for men vs. 26.7% for women) criteria. The most common metabolic disorder in this population was dyslipidaemia, regardless of the definition used. Substantial agreement, estimated using the kappa statistic, was found between the modified ATP III and IDF definition, whereas the lowest agreement was observed between the WHO and ATP III criteria.

**Conclusions:**

The MS is highly prevalent among Chinese adults in Pudong New Area of Shanghai and the most prevalent component was dyslipidemia. These findings underscore the importance of prevention and control efforts for the MS in this area and the need for a unified predictive definition for the syndrome for use by clinical practitioners and public health agencies.

## Background

The metabolic syndrome (MS) is a cluster of the most dangerous risk factors for type 2 diabetes mellitus and cardiovascular disease (CVD), including abdominal obesity, hypertension, hyperglycemia and dyslipidemia. When these metabolic abnormalities occur in the same individual they confer an additional cardiovascular risk above and beyond the contribution of the individual components [1,2]. It is estimated that the risk from the MS for major cardiovascular events is approximately twice as high as for those with the syndrome compared to those without it, and the risk for type 2 diabetes is around five-fold greater for those with the MS [2-4].

The MS is becoming epidemic around the world and the prevalence of the MS has been estimated to be more than 20%. The high prevalence of the MS has been reported not only in developed countries [5-7] but also in developing regions [8,9], including China [10-13]. China has experienced a remarkable economic expansion in the past three decades, and Pudong New Area of Shanghai is one of the most economically active areas in the country. This area of Shanghai has experienced a rapid nutrition transition characterized by an increasingly sedentary lifestyle and substantial dietary changes (e.g., increasingly energy-density) [14], as well as a dramatic aging of the population [15]. These changes are likely to have contributed to a high prevalence of the MS and increased risk for type 2 diabetes, CVD and premature death. In order to evaluate and predict the burden of CVD and other chronic diseases in this area, population-based estimates of the prevalence of the MS are needed.

So far, however, there has been no definition of the MS accepted universally. Currently, several definitions of the MS have been proposed for use, and each definition has a slightly different emphasis. While the one proposed by World Health Organization takes insulin resistance as a required component [16], the modified ATP III criteria treat the five components equally [17]. The IDF definition, on the other hand, puts an emphasis on central obesity [18]. Due to the lack of internationally agreed-upon criteria to define the condition, the prevalence of the MS has varied widely across different studies. Comparisons between the prevalence of the MS using these definitions may help better understand the characteristics of metabolic disorder among Chinese adults and facilitate interventions to prevent the MS and subsequent development of CVD.

In this study, we estimated and compared the prevalence of the MS by applying the three definitions of the condition in a representative sample of the residents in Pudong New Area of Shanghai, China.

## Methods

### Participants of the study

This cross-sectional study was conducted from April to July of 2008 among permanent residents of Pudong New Area of Shanghai, China, who had lived in the area for 5 years or more. A total of 6,387 eligible adults (20-79 yrs) were randomly selected from the area using a three-stage sampling design. Firstly, all 30 streets in this area were classified into three groups (each with 10 streets) according to the residents' average social economic status [14] and 12 streets (4 from each group) were randomly selected. Then, a total of 34 communities, which hold about 71,000 eligible residents, were randomly selected from the 783 communities in the selected streets. The expected number of participants in each community was calculated as 9.0% of its eligible population. Finally, a house number was randomly selected in each community as the first family interviewed. All eligible subjects in the selected families were recruited. Pregnant women and physically or mentally disabled persons were excluded from the survey. Of the 6,387 eligible adults contacted, 5,584 were interviewed and donated blood and urine samples, yielding a response rate of 87.4%. Of these participants, 2,477 were male and 3,107 were female, and 804 non-participants (12.6%) declined to be interviewed for miscellaneous reasons. The study was approved by Fudan University Institutional Review Board (IRB00002408, FWA00002399).

### Data collection

After obtaining written consent, a structured in-person interview was conducted by trained interviewers to elicit information about demographic factors, diagnosis of hypertension, diabetes and hyperlipidemia, tobacco and alcohol use, physical activity, and family history of hypertension and diabetes. At the interview, each participant was also measured for his/her blood pressure, body weight, standing height, and circumference of the waist and hip by trained staff. Blood pressure (BP) was measured on the right arm in the sitting position using standard mercury sphygmomanometer after at least 5 minutes of rest. The first and fifth Korotkoff sounds were recorded. Body height was measured to the nearest 0.1 cm by using a stadiometer. Girth measurements, recorded to the nearest 0.1 cm, were taken with a cloth tape. Waist circumference (WC) was measured at the midline between the lower border of the ribs and the iliac crest (usually at a level of 2.5 cm above the umbilicus) in the horizontal plane after a normal expiration. Hip circumference was defined as the maximum girth reading between waist and thigh. Body weight was measured with electronic scales to the nearest 0.1 kg. Two measurements were taken, with tolerances of <5 mmHg for blood pressure, <1 cm for height and circumference, and <1 kg for weight. A third measurement was taken if the difference between the first two exceeded the tolerance. The mean of the replicates was used in the following analyses. Body mass index (BMI: weight in kilograms divided by height in meters squared, kg/m^2^) and waist-to-hip circumference ratio (WHR) were calculated using the direct measurements.

### Laboratory measurements

All participants were asked to provide 10 ml fasting blood and 50 ml morning void urine for biochemical analysis in Shanghai Second People's Hospital. An automatic Biochemical Analyzer (HITACHI 7170A, Hitachi, Ltd, Tokyo, Japan) was used to measure the levels of fasting plasma glucose (FPG), triglycerides (TG), high-density lipoprotein cholesterol (HDL-C), low-density lipoprotein cholesterol (LDL-C) and total cholesterol (TC), as well as urinary concentrations of creatinine and albumin by using enzymology or immunoradiometry methods. Quality control of the assays was assessed internally and externally. The interassay coefficient of variation was <1.5% for FPG, <1.6% for TG, <3.0% for TC, <2.9% for HDL-C, <1.6% for LDL-C, <7.0% for urinary album, and <2.1% for urinary creatinine.

### Definitions of the metabolic syndrome

The WHO criteria [16] for the MS required the presence of diabetes mellitus or impaired fasting plasma glucose (FPG ≥ 5.6 mmol/L) and at least two of the following components: 1) diagnosed with hypertension or blood pressure: ≥ 140/90 mmHg; 2) dyslipidemia: TG ≥ 1.695 mmol/L or HDL-C ≤ 0.9 mmol/L (male), ≤ 1.0 mmol/L (female); 3) central obesity: WHR > 0.90 (male); > 0.85 (female), or BMI ≥ 30 kg/m^2^; or 4) microalbuminuria: urinary albumin to creatinine ratio (ACR) ≥ 30 mg/g.

The modified ATP III definition of the MS [17] required the presence of at least 3 or more of the following 5 components: 1) elevated WC: ≥ 90 cm (male), ≥ 80 cm (female); 2) elevated TG: > 150 mg/dL (1.695 mmol/L); 3) reduced HDL-C: < 1.036 mmol/L (male), < 1.295 mmol/L (female); 4) hypertension or elevated BP: ≥ 130/85 mmHg; or 5) diabetes or elevated FPG: ≥ 5.6 mmol/L.

The 2006 International Diabetes Federation (IDF) definition classified a person with the MS in the same way as ATP III, but required the presence of central adiposity (elevated WC) as an essential component [18].

### Statistical analysis

Statistical analysis was conducted using SAS (version 8.1; SAS Institute, Cary, NC). Chi-square and student *t *tests were used to compare the difference between categorical and numeric variables. Age-standardized prevalence of the MS was calculated based on the age distribution of the standard world population. Logistic regression models were fit to evaluate the association of individual metabolic abnormalities with the MS. In addition, kappa statistics were calculated to determine the level of agreement between the three MS definitions. P values < 0.05 were considered statistically significant.

## Results

### Measured metabolic characteristics of the study population

Table 1 presents the measured metabolic characteristics in our population. Compared to women, men had a significantly higher WC, WHR, BMI, higher systolic and diastolic BP and higher levels of FPG and TG. Men also had lower levels of HDL-C and ACR than women. The differences between men and women in prevalence of elevated BMI or WC were not significant, but men had a higher prevalence of elevated BP, FPG and TG, while women had a higher prevalence of low HDL-C and elevated ACR (Table 1).

**Table 1:** Measured metabolic characteristics of the selected Chinese adults in Pudong New Area of Shanghai, China

Characteristics	Male (N = 2477)	Female (N = 3107)	P value
*WC*			
Mean ± SD	83.9 ± 9.2	78.3 ± 9.4	<0.0001
≥ 90 cm for male	22.1		
≥ 80 cm for female		38.5	<0.0001
*BMI*			
Mean ± SD	24.1 ± 3.3	23.8 ± 3.5	<0.0001
≥ 30 kg/m^2^	4.5	5.3	0.16
*WHR*			
Mean ± SD	0.88 ± 0.06	0.83 ± 0.07	<0.0001
>0.90 for male	38.3		
>0.85 for female		37.9	0.93
*Systolic BP*			
Mean ± SD	128 ± 16.1	126 ± 19.6	<0.0001
≥ 130 mmHg	40.3	37.5	0.03
*Diastolic BP*			
Mean ± SD	81 ± 9.5	80 ± 10.1	<0.0001
≥ 85 mmHg	27.0	24.6	0.05
*FPG*			
Mean ± SD	5.8 ± 1.9	5.7 ± 1.6	0.0003
>5.6 mmol/l	35.9	31.4	0.0006
*HDL-C*			
Mean ± SD	1.34 ± 0.26	1.48 ± 0.27	<0.0001
<1.036 mmol/l for male	11.1		
<1.295 mmol/l for female		25.8	<0.0001
*TG*			
Mean ± SD	2.4 ± 2.1	2.1 ± 1.7	0.0001
>1.695 mmol/l	56.5	47.0	<0.0001
*Urinary ACR*			
Mean ± SD	2.0 ± 9.2	2.8 ± 11.1	<0.0001
≥ 30 mg/g	0.8	1.6	0.0035

P value for *t*-test (continuous variables) or χ^2 ^test (categorical variables).

### Sex and age-specific prevalence of the MS in the study population

The prevalence of the MS was observed to increase with increasing age in both sexes by either one of the three definitions. It is of note that the age-specific prevalence was higher in men than in women in early adulthood (before age of 65 according to WHO definition and at age of 40 using ATP III and IDF criteria) but appeared lower during elderly period (Table 2). The MS was more common in men (20.2%) than in women (18.7%) using WHO definition but was otherwise according to modified ATP III (28.4% for men vs. 35.1 for women) and IDF (15.9% for men vs. 26.7% for women) criteria.

**Table 2:** Sex and age-specific prevalence of the MS according to WHO, modified ATP III and IDF definition among selected Chinese adults in Pudong New Area of Shanghai, China

	No. of Subjects	WHO criteria		ATP III criteria		IDF criteria	
				
		No. of MS	%	No. of MS	%	No. of MS	%
*Male*							
	2477	498	20.2(15.3)	696	28.4(22.7)	391	15.9(13.8)
20-	141	3	2.1	10	7.1	9	6.4
25-	124	6	4.9	17	14.1	14	11.6
30-	134	8	6.1	16	12.2	12	9.2
35-	142	22	15.8	26	18.7	17	12.2
40-	204	35	17.2	54	26.7	34	16.8
45-	314	55	17.5	74	23.7	38	12.2
50-	309	76	19.6	114	29.5	61	15.8
55-	348	85	24.6	117	33.9	61	17.7
60-	258	80	31.3	103	40.2	51	19.9
65-	162	51	31.7	68	42.5	38	23.8
70-	173	44	25.4	62	35.8	34	19.7
75-	88	33	37.5	35	40.2	22	25.3
*Female*							
	3107	575	18.7(12.3)	1078	35.1(25.0)	821	26.7(19.2)
20-	159	0	0	3	1.9	3	1.9
25-	176	3	1.8	15	8.8	14	8.2
30-	156	3	1.9	13	8.4	10	6.5
35-	186	8	4.4	33	17.9	28	15.2
40-	274	20	7.4	48	17.8	34	12.6
45-	395	59	15.0	113	28.8	83	21.1
50-	551	98	17.9	205	37.6	149	27.3
55-	468	123	26.5	210	45.2	172	37.0
60-	265	80	30.2	147	55.5	110	41.5
65-	189	59	31.9	96	52.5	71	38.8
70-	188	75	39.9	130	69.5	99	52.9
75-	100	47	47.5	65	65.7	48	48.5

In parentheses was the age-standardized prevalence of the MS according to age distribution of the world population.

### Prevalence of individual metabolic abnormalities in the study population

The prevalence of individual metabolic abnormalities is presented in Table 3. According to WHO criteria, dyslipidemia, i.e. elevated TG or reduced HDL-C, was the most common metabolic abnormality in both men (56.9%) and women (47.8%), followed by central obesity, elevated FPG, raised BP and presence of microalbuminuria. Among the five metabolic abnormalities defined by modified ATP III or IDF criteria, elevated TG also ranked first in both sexes (59.2% for men and 49.7% for women), followed by elevated BP (49.0% for men and 44.8% for women), elevated FPG (36.7% for men and 32.2%), increased WC (22.1% for men and 38.5 for women) and reduced HDL-C (11.1% for men and 19.3% for women). In this population, the present of 1 or 2 metabolic abnormalities was most common in both men and women.

**Table 3:** Prevalence of individual metabolic abnormalities among selected Chinese adults in Pudong New Area of Shanghai, China


Metabolic abnormalities	Male (2477)		Female (3107)		Total (5583)	
			
	Number	%	Number	%	Number	%
*By WHO criteria*						
Impaired FPG or diabetes	903	36.7	992	32.2	1895	34.2
Central obesity	973	39.3	1224	39.4	2197	39.3
Elevated BP or hypertension	778	31.4	927	29.8	1705	30.5
Microalbuminuria	19	0.8	51	1.6	70	1.3
Hyperlipidemia	1409	56.9	1485	47.8	2894	51.8
No. of metabolic abnormalities						
0	483	19.6	859	27.9	1342	24.2
1	682	27.7	791	25.7	1473	26.6
2	675	27.4	696	22.6	1371	24.8
3	455	18.5	495	16.1	950	18.2
4	161	6.5	226	7.3	387	7.0
5	4	0.2	13	0.4	17	0.3
*By modified ATP III or IDF criteria*						
Increased WC	548	22.1	1196	38.5	1744	31.2
Elevated BP or hypertension	1213	49.0	1393	44.8	2606	46.7
Reduced HDL-C or use drug	272	11.1	795	25.8	1067	19.3
Elevated TG or use drug	1455	59.2	1532	49.7	2987	54.0
Impaired FPG or diabetes	903	36.7	992	32.2	1895	34.2
No. of metabolic abnormalities						
0	418	17.0	667	21.7	1085	19.6
1	663	27.0	677	22.0	1340	24.2
2	676	27.6	652	21.2	1328	24.0
3	460	18.8	557	18.1	1017	18.4
4	206	8.4	385	12.5	591	10.7
5	30	1.2	136	4.4	168	3.0

### Prevalence of the MS among subjects with a certain metabolic abnormality

**Figure 1:**
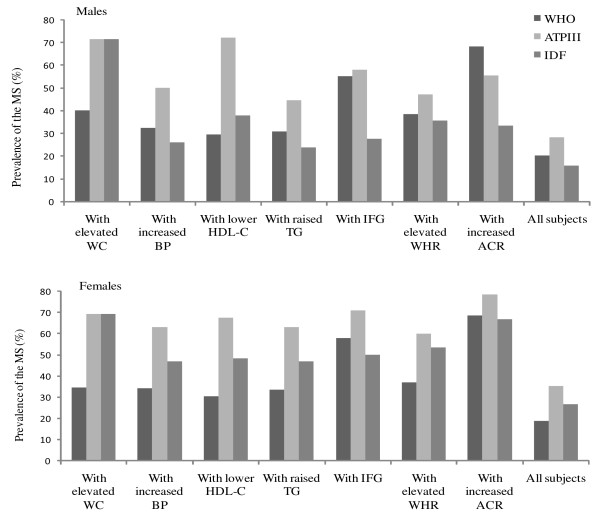
**Prevalence of the MS according to WHO, modified ATP III and IDF criteria in all participants and the subjects with each individual metabolic abnormality**.

Figure 1 shows the prevalence of MS estimated by each definition in all participants and among subjects with a specific metabolic abnormality. The prevalence of the MS was 40.0% for the men and 34.5% for the women with elevated WC using WHO definition, but the prevalence estimated was substantially greater when using the modified ATP III (71.6%) and IDF criteria (69.3%). Among the subjects with high BP, only 32.5% of the men and 34.1% of the women had the MS using the WHO definition whereas 50.0% of the men and 63.2% of the women were classified as such according to the modified ATP III definition. Difference in the prevalence of MS was also observed among subjects having other individual metabolic abnormalities.

### Association of individual metabolic abnormalities with the MS

We also examined the association of the individual metabolic abnormalities with the MS. Subjects with any one metabolic disorder were more likely to be diagnosed with the MS by modified ATP III definition (Table 4). Microalbuminuria, one component of the WHO criteria, was strongly associated with the condition in both sexes, but was not significantly related to the MS in men using the definitions of the ATP III or IDF. Interestingly, regardless of the definition used, subjects with elevated TG were most likely to be diagnosed with the MS, with odds ratios (OR) being 7.6, 15.9 and 5.1 for men and 7.3, 16.5 and 7.7 for women under WHO, ATPIII and IDF definition, respectively, after adjusting for age, BMI, alcohol consumption, cigarette smoking and exercise.

**Table 4:** Association of individual metabolic abnormalities with the MS among selected Chinese adults in Pudong New Area of Shanghai, China


Metabolic abnormalities	MS by WHO		MS by ATP III		MS by IDF	
			
	No/Yes	OR(95%CI)	No/Yes	OR(95%CI)	No/Yes	OR(95%CI)
*Male*						
Impaired FPG or diabetes						
No	1557/0	--	1379/176	1.0	1413/142	1.0
Yes	405/498	--	377/521	9.6(7.8-11.9)	648/250	3.5(2.8-4.4)
Central obesity (WC > 90 cm)						
No	1632/279	1.0	1602/305	1.0	1907/0	--
Yes	329/219	3.9(3.1-4.8)	154/392	14.9(11.8-18.9)	154/392	--
Hypertension or elevated BP (sbp ≥ 130 or dbp ≥ 85 mmHg)						
No	1143/107	1.0	1151/96	1.0	1170/77	1.0
Yes	819/391	4.1(3.2-5.2)	605/601	10.7(8.4-13.7)	891/315	5.1(3.9-6.7)
Microalbuminuria						
No	1956/485	1.0	1748/687	1.0	2049/386	1.0
Yes	6/13	6.8(2.5-18.7)	8/10	2.4(0.9-6.1)	12/6	2.1(0.8-5.8)
Reduced HDL-C or use drug^a^						
No	1770/416	1.0	1681/502	1.0	1893/290	1.0
Yes	192/80	1.5(1.1-2.0)	75/195	9.9(7.2-13.8)	168/102	3.2(2.3-4.6)
Elevated TG or use drug^a^						
No	950/50	1.0	950/49	1.0	953/46	1.0
Yes	1009/446	7.6(5.5-10.4)	806/648	15.9(11.3-22.2)	1108/346	5.1(3.5-7.4)
Dyslipidemia (reduced HDL-C or elevated TG)^a^						
No	1007/60	1.0	986/76	1.0	1001/61	1.0
Yes	955/438	7.4(5.5-10.0)	770/621	10.9(8.2-14.6)	1060/331	4.2(3.0-5.8)
*Female*						
Impaired FPG or diabetes						
No	2088/0	--	1706/380	1.0	1757/329	1.0
Yes	417/575	--	288/700	8.4(7.0-10.1)	495/493	3.9(3.3-4.7)
Central obesity (WC > 85 cm)						
No	1427/166	1.0	1631/258	1.0	1889/0	--
Yes	778/409	4.0(3.3-4.9)	363/822	12.0(10.0-14.5)	363/822	--
Hypertension or elevated BP (sbp ≥ 130 or dbp ≥ 85 mmHg)						
No	1590/103	1.0	1484/205	1.0	1515/174	1.0
Yes	915/472	4.9(3.8-6.2)	510/875	8.3(6.8-10.0)	737/648	5.3(4.3-6.5)
Microalbuminuria						
No	2489/540	1.0	1983/1040	1.0	2235/788	1.0
Yes	16/35	7.8(4.0-15.3)	11/40	5.3(2.5-11.2)	17/34	4.1(2.2-7.8)
Reduced HDL-C or use drug^a^						
No	1951/333	1.0	1738/543	1.0	1841/440	1.0
Yes	553/241	2.3(1.8-2.8)	256/537	10.4(8.2-13.1)	411/382	4.0(3.2-5.0)
Elevated TG or use drug^a^						
No	1485/62	1.0	1432/115	1.0	1440/107	1.0
Yes	1017/511	7.3(5.4-9.7)	562/965	16.5(12.9-21.2)	812/715	7.7(6.0-9.9)
Dyslipidemia (reduced HDL-C or elevated TG)^a^						
No	1547/73	1.0	1456/161	1.0	1475/142	1.0
Yes	958/502	7.1(5.4-9.4)	538/919	12.1(9.7-15.2)	777/680	5.9(4.7-7.5)

OR: Adjusted for age as a continuous variable.

^a ^additionally adjusted for BMI (as a continuous variable), cigarette smoking (Never/Ever), alcohol consumption (Never/Ever) and exercise (Never/ever).

### Agreement between the three MS definitions

**Figure 2:**
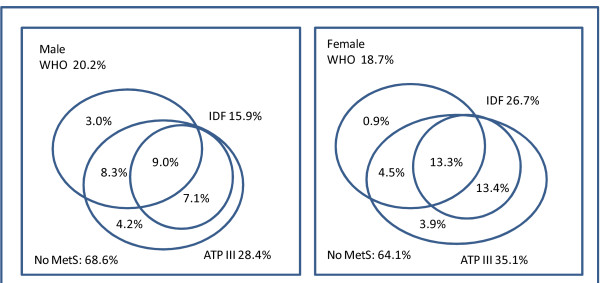
**Overall prevalence of the MS among selected Chinese adults in Pudong New Area of Shanghai, China, according to WHO, modified ATP III and IDF criteria**.

Figure 2 shows the overall prevalence of the MS by WHO, modified ATP III and IDF criteria. Nine percent of men and 13.3% of women were consistently classified as having the MS by each definition and 68.6% of men and 64.1% of women were classified as being free from the MS consistently. The level of agreement between each two definitions was modest, with kappa value ranging from 0.382 to 0.806 (Table 5). Agreement between modified ATP III and IDF criteria was substantial with a kappa of 0.647 for men and 0.806 for women. All subjects classified with the MS by the IDF definition were also classified this way by the ATP III criteria. The lowest level of agreement was between the WHO and IDF definitions in both men (κ = 0.382) and women (κ = 0.469).

**Table 5:** Agreement of WHO, modified ATP III and IDF criteria in identifying the MS among selected Chinese adults in Pudong New Area of Shanghai, China

	WHO criteria		κ	95%CI	ATP III criteria		κ	95%CI
						
	+	-			+	-		
*Male*								
ATP III criteria								
+	434	276			--	--		
-	73	1670	0.617	0.611-0.622	--	--	--	
IDF criteria								
+	220	177			397	0		
-	287	1769	0.382	0.377-0.387	313	1743	0.647	0.643-0.652
*Female*								
ATP III criteria								
+	553	532			--	--		
-	27	1962	0.552	0.547-0.558	--	--	--	
IDF criteria								
+	413	414			827	0		
-	167	2080	0.469	0.464-0.474	258	1989	0.806	0.802-0.810

P < 0.01 for all κ statistics.

## Discussion

In this representative sample of adults whose age and sex distribution was similar to that in Pudong New Area of Shanghai, China [19], we found that the MS was highly prevalent. Using the modified criteria of ATP III for the MS the prevalence was 28.4% for men and 35.1% for women. The overall and age-specific prevalence of the MS in this population were much higher than the general levels of Chinese adults in 1992 and 2000-01 [20,21], and were similar to those in other economically developed areas in China [11,13]. In fact, the prevalence of the MS was comparable to those in developed countries [5,22].

Consistent with the results from studies in other populations [5,13,22], the prevalence of the MS increased with age in both sexes in our study. We observed a wide discrepancy between the crude and age-standardized prevalence of the MS, indicating that the increasing proportion of older adults in the population has contributed much to the overall prevalence of the MS in this area. The number of adults at age 60+ years in Shanghai reached 3.0 million in 2008, and they account for more than 20 percent of all residents in the city [23]. Interestingly, in early adulthood the age-specific prevalence was higher in men than in women but this pattern was reversed among older adults. The age-specific prevalence pattern we observed was consistent with that in a Japanese population [24], but differed from several other previous studies, in which a higher age-specific prevalence of the MS was consistently observed for women [5,10,11]. The explanation for this finding is not immediately clear, and the pattern varied by the definition employed. It is possible that the men in Shanghai were at a higher risk of the MS than women, and thus had a higher mortality from CVD and diabetes in later adulthood. In addition, selection biases and chance cannot be excluded. Notably, the cross-over point of the sex specific prevalence estimates by age was much later in life when the WHO definition was used (about age 65), compared to the modified ATP III or IDF criteria (about age 40). This may explain the higher overall prevalence of the MS in men using WHO but the higher prevalence estimates in women according to modified ATP III and IDF criteria.

Another significant finding in this study is that dyslipidemia, particularly hypertriglyceridemia, is the most common metabolic disorder in our population. This finding is much different from those of a national estimate from all of China [10], even after taking age differences and the MS definitions used in the two studies into account. The differences in MS components may be due to differences in behavioral risk factors (i.e. diet or lifestyle) rather than genetic susceptibility. The rapid "nutrition transition" in Shanghai, characterized by a more sedentary lifestyle and an energy-dense higher fat diet may, at least in part, account for the higher prevalence of hyperlipidemia in our population. This element of the "nutrition transition" is a central contributor of hyperlipidemia [25] and may function as initiation factor for the MS [26].

Importantly, the distinct characteristic of the metabolic disorder in this population may indicate predictive value of the MS for the risk of CVD and other chronic diseases in the area. The definitions of the MS, either proposed by WHO, ATP III or IDF, similarly focus on obesity, hypertension, hyperglycaemia and dyslipidemia. The MS is usually identified by the presence of at least 3 of 5 metabolic abnormalities. It is thus plausible that the characteristics of the MS vary across populations. For example, blacks in the US are more likely than the whites to have the clustering of obesity, hypertension and diabetes but less likely to have either elevated TG or low HDL-C levels, leading to a lower prevalence of the MS but higher rates of diabetes and CVD in the blacks than in the whites [27]. If it is true that dyslipidemia is less important in predicting the diabetes and CVD than other metabolic disorders, as suggested by Sumner *et al*'s [27], the fact that dyslipidemia is the most common metabolic abnormality in our population may indicate and account for the comparable prevalence of the MS but a lower incidence of diabetes and CVD in Chinese adults than in their western counterparts.

The level of agreement we observed among the three definitions was modest and not surprisingly the prevalence of the MS was quite different according to each individual definition employed. This finding is consistent with previous studies conducted in Chinese adults [13,21], and other populations [6,7,28-30]. Although 68.6% of the men and 64.1% of the women were consistently classified as being free of MS by all 3 definitions, only 9.0% of men and 13.3% of women were consistently classified with the MS by all three criteria. More than 20% of the participants were classified differently using the three definitions. In addition, the prevalence of the MS varied greatly among the subjects with specific individual metabolic abnormalities depending on the definition employed. This reflects that the three definitions have their own respective emphasis. The WHO definition directly assigns greatest value to insulin resistance which is usually indicated by impaired fasting glucose. In contrast, the modified ATP III and IDF definitions do not employ insulin resistance as a required component based on the assumption that the other four components of the MS are linked to insulin resistance. However, significant proportions of those with the four individual components in our sample did not evidence of have insulin resistance as indicted by the criteria employed. For example, there is evidence suggesting no strong link between hypertension and insulin resistance [31]. The phenomenon may explain the discrepancy in the prevalence of the MS in the same population estimated by three definitions. A single worldwide definition of the MS would be desirable to make comparisons between different populations easier and for clinical, epidemiologic and surveillance purposes. There is a need for existing cohort studies in Asia to explore the best and most predictive definition of the MS and its components.

An important limitation of the present study is the cross-sectional design. This fact limited our ability to examine the most predictive MS definition for the risk of diabetes, CVD and premature death among Chinese adults. Moreover, the prevalence of the MS by WHO criteria might be underestimated in this study because impaired fasting glucose was used as the surrogate of impaired glucose tolerance which is more common in Chinese adults [32]. Finally, we cannot exclude problems associated with selection bias from the study, although our response rate to the study was quite high (89.4%). The strengths of this study include the representative sample population, large sample size, and our rigorously standardized methods for measuring the MS components.

## Conclusion

In summary, this cross-sectional study shows that the MS characterized with dyslipidemia is highly prevalent in adult population in Pudong New Area of Shanghai, China. The ongoing nutrition transition in this area and the aging of the population may have caused and will aggravate this problem in future years. Given the relatively high prevalence estimates regardless of the definition used, there is a need for a unified definition of the MS in Chinese adults that may guide prevention and treatment efforts to fight against the growing MS epidemic in China.

## Abbreviations

MS: metabolic syndrome; WHO: World Health Organization; NCEP-ATP III: National Cholesterol Education Program Adult Treatment Panel; IDF: International Diabetes Federation; CVD: cardiovascular diseases; BP: Blood pressure; WC: waist circumference; BMI: Body mass index; WHR: waist-to-hip circumference ratio; FPG: fasting plasma glucose; TG: triglycerides; HDL-C: high-density lipoprotein cholesterol; LDL-C: low-density lipoprotein cholesterol; TC: total cholesterol; ACR: albumin to creatinine ratio.

## Competing interests

The authors declare that they have no competing interests.

## Authors' contributions

WHX contributed to the analysis and interpretation of the data and drafting of the manuscript. XNR, QLZ, HZ, YZ and HQ had contribution in collection, management and analysis of the study data. XJF, YB, HYW, QS and LMY contributed to the organization of the field work and collection of the study data. GMZ, JJG and QWJ contributed to the study concept and design, collection of the study data and critical revision of the article. All authors read and approved the final version of the manuscript.

## Pre-publication history

The pre-publication history for this paper can be accessed here:

http://www.biomedcentral.com/1471-2458/10/246/prepub
